# Characterization of nano α-TCP: Setting time, antibacterial activity and cytotoxicity on fibroblast cells

**DOI:** 10.1016/j.jtumed.2026.05.009

**Published:** 2026-06-05

**Authors:** Nadya L. Riany, Selviana Wulansari, Ade P. Dwisaptarini, Moehamad O. Roeslan, Thalia C. Maharani, Faradilla J. Azzahra

**Affiliations:** a Faculty of Dentistry, Universitas Trisakti, Jakarta, Indonesia; b Department of Conservative Dentistry, Faculty of Dentistry, Universitas Trisakti, Jakarta, Indonesia; c Department of Oral Biology, Faculty of Dentistry, Universitas Trisakti, Jakarta, Indonesia

**Keywords:** فوسفات ألفا ثلاثي الكالسيوم النانوي, مضاد للبكتيريا, العقدية الطافرة, السمية الخلوية, اختبار عدّ الخلايا-8, الخلايا الليفية, Antibacterial, CCK-8 assay, Cytotoxicity, Fibroblasts, Nano α-tricalcium phosphate, *Streptococcus mutans*

## Abstract

**Background:**

Nano α-Tricalcium Phosphate (nano α-TCP) is a potential pulp capping material due to its calcium/phosphate ion release, antibacterial properties, and biocompatibility.

**Objectives:**

To evaluate the setting time, antibacterial activity against *Streptococcus mutans*, and cytotoxicity of nano α-TCP.

**Methods:**

Setting time was measured using the Vicat test. Antibacterial activity was assessed by direct contact testing at concentrations of 100%, 50%, and 25%. Cytotoxicity on fibroblasts was evaluated using the CCK-8 assay after 24 and 72 hours.

**Results:**

Nano α-TCP at 100% concentration showed the strongest antibacterial effect and the fastest setting time. All tested concentrations maintained high fibroblast viability, indicating low cytotoxicity.

**Conclusion:**

Nano α-TCP effectively inhibits *S. mutans*, sets rapidly, and is biocompatible, supporting its potential as a pulp capping material.

## الملخص

**أهداف البحث:**

يُعد فوسفات ألفا ثلاثي الكالسيوم النانوي مادة مبتكرة يُنظر إليها على أنها مناسبة لتغطية اللب، نظرًا لقدرتها على إطلاق أيونات الكالسيوم والفوسفات، مما يؤدي إلى تكوين بيئة قلوية تعزز تكوّن العاج الترميمي، وتحد من نمو البكتيريا، وتسرّع عملية التصلّب. وبالإضافة إلى النشاط المضاد للميكروبات، يُعد التوافق الحيوي عاملًا أساسيًا في مواد تغطية اللب، لأنها تكون على تماس مباشر مع نسيج اللب. لذلك، هدفت هذه الدراسة إلى تقييم زمن التصلّب، والنشاط المضاد للبكتيريا ضد العقدية الطافرة، والسمية الخلوية لفوسفات ألفا ثلاثي الكالسيوم النانوي في الخلايا الليفية.

**طرق البحث:**

قِيس زمن التصلّب باستخدام اختبار فيكات، وقُيّم النشاط المضاد للبكتيريا باستخدام اختبار التلامس المباشر بتراكيز 100% و50% و25% من فوسفات ألفا ثلاثي الكالسيوم النانوي. استُخدم مجمّع ثلاثي أكسيد المعادن وهيدروكسيد الكالسيوم كضوابط موجبة، والماء المقطر كضابط سالب. وحُدد النشاط المضاد للبكتيريا بناءً على نمو العقدية الطافرة على الآغار. كما اختُبرت السمية الخلوية في الخلايا الليفية باستخدام اختبار عدّ الخلايا-8 بتراكيز 100 و50 و25 ميكروغرام/مل من فوسفات ألفا ثلاثي الكالسيوم النانوي. وقِيسَت حيوية الخلايا بعد 24 و72 ساعة باستخدام مقياس الطيف الضوئي.

**النتائج:**

أظهر فوسفات ألفا ثلاثي الكالسيوم النانوي بتركيز 100% أعلى تأثير مضاد للبكتيريا، إذ لم يُلاحظ أي نمو بكتيري، كما أظهر أسرع زمن تصلّب. وفي اختبارات السمية الخلوية، أدت جميع التراكيز المختبرة من فوسفات ألفا ثلاثي الكالسيوم النانوي إلى حيوية أعلى للخلايا الليفية مقارنة بالضابط السالب، مما يشير إلى انخفاض السمية الخلوية.

**الاستنتاجات:**

كان فوسفات ألفا ثلاثي الكالسيوم النانوي فعالًا في تثبيط نمو العقدية الطافرة وتسريع زمن التصلّب، لا سيما عند التراكيز الأعلى. كما لم يُظهر سمية تجاه الخلايا الليفية، مما يؤكد إمكانية استخدامه مادةً متوافقة حيويًا لتغطية اللب.

## Background

Dental caries remains one of the most prevalent oral diseases worldwide and a major cause of hard tissue destruction in teeth. According to the 2023 Indonesian Health Survey, the prevalence of dental caries among individuals aged three and older was 82.8%.^1^ Caries develops through tooth structure demineralization due to acid production by bacterial metabolism, predominantly that of *Streptococcus mutans*.^2^ This pathological process begins with the formation of dental plaque as a complex biofilm that provides a favorable environment for acidogenic bacteria.^3^ Without appropriate intervention, demineralization can extend into the dentin and pulp chamber, leading to inflammation, infection, and pulp necrosis.^4^ Under these conditions, preserving pulp vitality is critically important because the dental pulp contains cells, blood vessels, and nerve tissues that are essential for maintaining vitality of the pulp.^3^^,^^4^

A clinical procedure used to preserve pulpal vitality is direct pulp capping, which involves applying a bioactive material directly onto the exposed pulp tissue to stimulate the formation of reparative dentin.^5^^,^^6^ The materials used for this procedure are expected to exhibit excellent biocompatibility, antibacterial properties, the ability to promote odontogenic potential, and dimensional stability.^7^ Calcium hydroxide (Ca(OH)_2_) has long been considered the standard material for direct pulp capping due to its ability to elevate the pH through the release of hydroxyl ions.^8^ In addition, mineral trioxide aggregate (MTA), a calcium silicate based material, is widely acknowledged due to its superior biocompatibility and favorable outcomes in pulp tissue regeneration.^9^^,^^10^ However, despite these advantages, both materials have considerable drawbacks. In particular, calcium hydroxide has poor handling properties in moist environments and a prolonged setting time, and MTA is limited by its long setting time and high cost.^8^ Consequently, nano α-tricalcium phosphate (nano α-TCP) has recently emerged as a potential bioactive alternative for direct pulp capping applications. α-TCP is a form of calcium phosphate with the ability to stimulate hard tissue formation.^11^ Previous studies have demonstrated the bioactivity of α-TCP, but limitations remain in terms of inconsistent setting kinetics, insufficient antibacterial evaluations, and the lack of comprehensive cytotoxicity assessments, particularly for nanoscale formulations.^12^^,^^13^

When produced at the nanoscale, α-TCP has an expanded surface area that significantly enhances its chemical reactivity and facilitates faster hydration when combined with water-based solutions^11^^,^^14^ This process releases calcium and phosphate ions to promote the formation of hydroxyapatite crystals, increase the local pH, and create an unfavorable environment for the growth of bacteria such as *Streptococcus mutans*.^11^^,^^14^ Furthermore, the high reactivity of nano α-TCP accelerates the setting time and improves the mechanical properties of the material compared with the micron-sized form.^15^ In addition to the antibacterial activity and setting time, it is important to evaluate the biocompatibility of nano α-TCP based on cytotoxicity assays. A commonly used method involves assessing the effect of a material on fibroblast cells. Fibroblasts are used as a standard model for cytotoxicity testing rather than primary cells responsible for reparative dentin formation.^16^, ^17^, ^18^ Cytotoxicity assays aim to confirm that a material is not toxic to cells and safe for clinical applications. Based on the considerations above, the aims of the present study were to evaluate the characteristics of nano α-TCP, including the setting time, antibacterial effectiveness against *Streptococcus mutans*, and cytotoxicity toward fibroblast cells.

## Materials and Methods

This in vitro experimental study was conducted in the laboratory using a post-test only control group design. The study was conducted from October to November 2024 at the Dental Materials and Testing Center, Faculty of Dentistry, Universitas Trisakti, and the Integrated Research Laboratory, YARSI University, Indonesia.

### Preparation of nano α-TCP

Calcium carbonate (CaCO_3_, ≥99%, Sigma–Aldrich, USA) and dicalcium phosphate anhydrous (CaHPO_4_, ≥98%, Merck, Germany) were used as precursors for the synthesis of nano α-TC.

Nano α-TCP was synthesized using a high-energy ball milling method with 8 mm zirconia balls for 120 min in a high energy milling system (SPEX 8000M Mixer/Mill, SPEX SamplePrep, Metuchen, NJ, USA). The resulting particles had an average size of 78.9 nm, and within the nanoscale range (1–100 nm).

The α-TCP powder was mixed with distilled water at a defined liquid-to-powder ratio to form a homogeneous paste. The concentrations of 100%, 50%, and 25% refer to the dilution levels of the prepared paste using sterile distilled water.

### Setting time test

The setting time was evaluated using a calibrated Vicat apparatus. Nano α-TCP samples prepared at concentrations of 100%, 50%, and 25% were placed into cylindrical metal molds (10 mm in diameter and 2 mm in height). The surface was leveled prior to testing.

Penetration measurements were performed at intervals of 5 min until the needle failed to produce a visible indentation on the sample surface. The final setting time was recorded as the time elapsed from initial placement until no indentation was observed.

### Antibacterial activity against *Streptococcus mutans*

a. Bacterial culture and medium

Brain heart infusion (BHI) agar and broth (Oxoid Ltd, Basingstoke, UK) were used as culture media. *Streptococcus mutans* (ATCC 25175, American Type Culture Collection, Manassas, VA, USA) was cultured in BHI broth and adjusted to McFarland standard 0.5 (1.5 × 10^8^ colony-forming units per milliliter (CFU/mL)).

b. Direct contact test

Nano α-TCP samples at concentrations of 100%, 50%, and 25%, MTA (ProRoot® MTA, Dentsply Sirona, Tulsa, OK, USA), calcium hydroxide (Dycal®, Dentsply Sirona, Milford, DE, USA), and distilled water (negative control) were placed into 96-well plates to a height of approximately 2 mm.

Next, 10 μL of bacterial suspension was added to each well and allowed to contact the material for 1 h. Subsequently, 215 μL of BHI broth was added, before incubating at 37 °C for 24 h. Serial dilutions (10^1^, 10^3^, and 10^5^) were prepared, and 10 μL of the 10^5^ dilution was plated onto BHI agar. The plates were incubated for an additional 24 h, and bacterial colonies were quantified as CFU/mL using the plate count method.

### Cytotoxicity assay using fibroblast cells

Fibroblast cells were cultured in Dulbecco's modified Eagle medium (Gibco®, Thermo Fisher Scientific, Waltham, MA, USA) supplemented with 1% penicillin–streptomycin (Gibco®, Thermo Fisher Scientific, Waltham, MA, USA) and 20% fetal bovine serum (Gibco®, Thermo Fisher Scientific, Waltham, MA, USA). Cells were incubated at 37 °C in a humidified atmosphere containing 5% CO_2_ until approximately 80% confluence was reached.

Cells were harvested using phosphate-buffered saline (Gibco®, Thermo Fisher Scientific, Waltham, MA, USA) and 0.25% trypsin–EDTA (Gibco®, Thermo Fisher Scientific, Waltham, MA, USA), followed by centrifugation at 5000 rpm and 25 °C for 5 min. Cells were seeded in 96-well plates at a density of 1 × 10^4^ cells per well and incubated for 24 h. The experimental treatments consisted of nano α-TCP at concentrations of 100, 50, and 25 μg/mL, with MTA (ProRoot® MTA, Dentsply Sirona, Tulsa, OK, USA) as the positive control and untreated cells as the negative control.

Cytotoxicity was evaluated using a Cell Counting Kit-8 (CCK-8, Dojindo Molecular Technologies, Kumamoto, Japan). The culture medium was replaced with 100 μL of a mixture of CCK-8 reagent and complete medium, before incubating for 1 h and continuing incubation up to 72 h. The absorbance was measured at 450 nm using a microplate spectrophotometer and the results were converted into cell numbers based on a standard curve.

### Statistical analysis

Statistical analyses was performed using GraphPad Prism version 9.0 (GraphPad Software Inc., San Diego, CA, USA). Data normality was assessed using the Shapiro–Wilk test. Normally distributed data (*p* > 0.05) were subjected to two-way analysis of variance followed by Tukey's post hoc test. The Kruskal–Wallis test followed by Dunnett's T3 post hoc test was conducted for non-normally distributed data (*p* < 0.05). A *p*-value <0.05 was considered to indicate a statistically significant difference.

## Results

According to the setting time test results, significant differences were observed among the experimental treatments. Overall, the setting time was shortest for 100% nano α-TCP and longest for 25% nano α-TCP. These findings indicate that higher concentrations of nano α-TCP promoted a faster hydration reaction, resulting in a shorter setting time (Table 1).

**Table 1:** Mean setting times (min) under different treatments.

Treatment	Mean time (min) ± standard deviation
Nano α-TCP 100%	60.33 ± 2.52
Nano α-TCP 50%	75.33 ± 3.12
Nano α-TCP 25%	85.33 ± 4.05
MTA (positive control)	71.00 ± 2.80
Ca (OH)_2_ (positive control)	63.33 ± 2.45

The antibacterial test results demonstrated that there were marked differences among the treatments. Bacterial growth by *Streptococcus mutans* did not occur under 100% nano α-TCP and the positive control materials (MTA and Ca(OH)_2_). By contrast, the number of bacterial colonies gradually increased under 50% and 25% nano α-TCP. Very high bacterial growth was found under the negative control (distilled water) (Table 2).

**Table 2:** *Streptococcus mutans* counts (CFU/mL) under different treatments.

Treatment	Mean colony count (CFU/mL)	Replicates
Nano α-TCP 100%	0	4
Nano α-TCP 50%	3075 × 10^5^	4
Nano α-TCP 25%	22,100 × 10^5^	4
MTA (positive control)	0	4
Ca (OH)_2_ (positive control)	0	4
Distilled water (negative control)	49,875 × 10^5^	4

**Figure 1:**
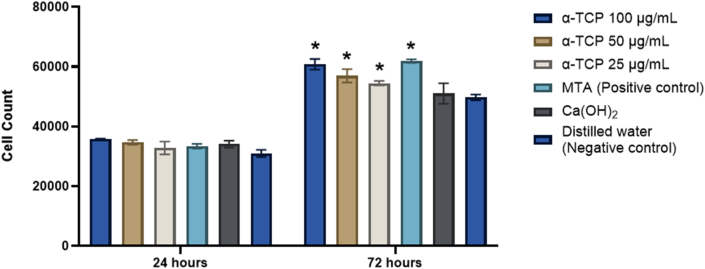
Cell counts obtained in CCK-8 cytotoxicity assays under different treatments and control treatments after exposure for 24 and 72 h. Asterisks (∗) indicate statistically significant differences compared with the negative control at the same time point (*p* < 0.05).

The CCK-8 method was employed for cytotoxicity testing and the results were recorded using a spectrophotometer at a wavelength of 450 nm. The cell numbers obtained from the cytotoxicity assays are presented in Figure 1.

After 24 h, the cell count under the negative control was 30,192 ± 1846.17. The cell counts under the nano α-TCP treatments were 35,636 ± 396.59 at 100 μg/mL, 34,608 ± 668.72 at 50 μg/mL, and 32,652 ± 1752.36 at 25 μg/mL. The cell count under the positive control (MTA) was 32,534 ± 1819.14, and that under Ca(OH)_2_ was 33,635 ± 1316.21.

After 72 h, the cell count under the negative control was 50,178 ± 1221.79. The cell counts under the nano α-TCP treatments were 60,012 ± 2148.68 at 100 μg/mL, 57,254 ± 1938.81 at 50 μg/mL, and 54,825 ± 906.73 at 25 μg/mL. The cell count under the positive control (MTA) was 61,287 ± 1225.80, and that under Ca(OH)_2_ was 51,812 ± 3225.87. Figure 1 shows that compared with the negative control, nano α-TCP at concentrations of 100, 50, and 25 μg/mL had no cytotoxic effects on fibroblast cells after exposure for 24 or 72 h.

## Discussion

This study aimed to evaluate the potential of using nano α-TCP as an alternative material for direct pulp capping by assessing three main parameters: setting time, antibacterial activity against *Streptococcus mutans*, and cytotoxicity toward fibroblast cells. The use of nano α-TCP is based on its unique characteristics, including its ability to release calcium and phosphate ions, and nanoscale particle size, which provides a high surface area and greater reactivity compared with its conventional form.^19^

### Setting time of nano α-TCP

The present study demonstrated that nano α-TCP at a concentration of 100% had the shortest setting time (60.33 min), followed by MTA (71.00 min) and lower concentrations of nano α-TCP. These findings are consistent with previous reports that nanoscale calcium phosphate materials exhibit enhanced reactivity due to their increased surface area, which accelerates hydration reactions and phase transformation into hydroxyapatite.^20^

The accelerated setting behavior of nano α-TCP can be attributed to its higher surface energy and increased contact area with the liquid phase, facilitating rapid dissolution–precipitation processes. During hydration, α-TCP reacts with water to form calcium-deficient hydroxyapatite, which contributes to matrix hardening and structural integrity. Recent studies have confirmed that nanosized calcium phosphate particles significantly improve the reaction kinetics compared with their microscale counterparts.^21^

However, although nano α-TCP had a shorter setting time than MTA, the clinical significance of this difference (≈11 min) may be limited and requires further investigation. In clinical practice, both materials still fall within a relatively long setting time range, and further optimization may be required to achieve a clinically meaningful improvement.

### Antibacterial activity

The antibacterial test showed that nano α-TCP at a concentration of 100% completely inhibited the growth of *Streptococcus mutans*, and its performance was comparable to both MTA and calcium hydroxide. Lower concentrations of nano α-TCP (50% and 25%) had reduced antibacterial activities, indicating a concentration-dependent effect.

The antibacterial mechanism of nano α-TCP is multifactorial. One of the primary mechanisms involves the release of calcium (Ca^2+^) and phosphate (PO_4_^3−^) ions during hydration and degradation, which can increase the local pH and create an unfavorable environment for bacterial survival.^22^ Elevated pH levels are known to disrupt bacterial cell membranes, denature proteins, and interfere with enzymatic activities, ultimately leading to bacterial cell death. Recent studies have shown that calcium phosphate-based biomaterials can exert antibacterial effects through ion release and local alkalization.^23^^,^^24^

In addition, the nanoscale size of α-TCP particles contributes to increasing the surface area and enhancing interactions with bacterial cells, which may facilitate direct contact-mediated antibacterial effects, further inhibiting bacterial adhesion and proliferation. Previous studies have also reported that the surface properties and size of particles play critical roles in antibacterial effectiveness.^25^

Despite these promising findings, calcium and phosphate ion release or pH changes over time were not directly measured in the present study. Therefore, the proposed antibacterial mechanisms remain hypothetical and should be confirmed in future studies through quantitative analysis of ion release kinetics and pH variations.

### Cytotoxicity and biocompatibility

The cytotoxicity results demonstrated that the nano α-TCP treatments at all test concentrations (100, 50, and 25 μg/mL) had no toxic effects on fibroblast cells, and they actually supported cell proliferation over time, thereby indicating the good biocompatibility of nano α-TCP and agreeing with previous findings that calcium phosphate materials are generally well tolerated by mammalian cells.

The favorable cytocompatibility of nano α-TCP can be attributed to its chemical similarity to natural bone mineral and capacity to release bioactive ions that promote cellular responses. In particular, calcium ions play crucial roles in regulating cell signaling pathways involved in proliferation, differentiation, and tissue repair. Several studies have demonstrated that calcium phosphate materials can enhance fibroblast proliferation and support tissue regeneration processes.^26^^,^^27^

It is important to note that the CCK-8 assay used in this study measures cellular metabolic activity rather than absolute cell numbers. Therefore, increased absorbance values should be interpreted as an indication of enhanced metabolic activity by viable cells rather than a direct measure of cell proliferation. This distinction is critical to avoid over-interpreting the results.

Furthermore, fibroblasts were selected as a standard model for cytotoxicity testing due to their reproducibility and sensitivity to biomaterials.^28^ However, they are not fully representative of the odontoblast-like cells responsible for dentin formation. Thus, future studies should test dental pulp stem cells or odontoblast-like cells to better evaluate the regenerative potential of nano α-TCP in pulp capping applications.

### Relationship between properties

It is important note that the properties evaluated in this study, i.e., the setting time, antibacterial activity, and cytotoxicity, were assessed at different concentrations. The antibacterial and setting time tests were conducted using bulk material concentrations (100%, 50%, and 25%), whereas cytotoxicity was evaluated at significantly lower concentrations (25–100 μg/mL). Thus, it cannot be concluded that the optimal antibacterial properties, fastest setting time, and highest biocompatibility were obtained at the same concentration. This limitation highlights the need for future studies to establish a clinically relevant concentration that simultaneously optimizes all of these key properties.

### Study limitations and future perspectives

The present study had several limitations that should be acknowledged. First, the physicochemical characterization of nano α-TCP was limited to particle size analysis, without conducting detailed evaluations of morphology, phase composition, crystallinity, or surface area, and thus advanced techniques such as scanning electron microscopy, transmission electron microscopy, X-ray diffraction, Fourier transform infrared spectroscopy, and Brunauer, Emmett, and Teller analysis are required for further comprehensive characterization. Second, the antibacterial mechanism was not directly investigated because the ion release kinetics or pH changes were not measured despite their assumed roles. Third, the biological evaluations were only conducted under in vitro conditions, which are not fully representative of the complex dental pulp environment, highlighting the need for in vivo validation. In addition, differences in the concentration ranges applied in the antibacterial/setting tests and cytotoxicity assays limit direct comparison of the results.

Furthermore, cytotoxicity assessment was restricted to fibroblast cells, which are not fully representative of the behavior of odontoblasts or dental pulp stem cells, indicating the need for more clinically relevant models such as human dental pulp stem cells. The antibacterial evaluation was limited to a single bacterial species, whereas clinical infections are polymicrobial, warranting the use of multispecies biofilm models in future studies. In addition, important functional properties such as the sealing ability, mechanical strength, solubility, and long-term stability were not assessed. Despite these limitations, this study provides preliminary evidence supporting the potential use of nano α-TCP as a bioactive material for restorative dentistry, which should be further validated in comprehensive physicochemical and biological investigations.

## Conclusion

Nano α-TCP exhibited promising characteristics as a potential bioactive material for use in direct pulp capping applications. Higher concentrations obtained faster setting times and enhanced antibacterial activities against *Streptococcus mutans*. In addition, nano α-TCP exhibited good biocompatibility, where all of the test concentrations (25–100 μg/mL) had no cytotoxic effects and supported fibroblast cell proliferation. However, a single optimal concentration that simultaneously maximized the antibacterial efficacy, setting performance, and biological safety was not determined in this study. Therefore, higher concentrations appear advantageous in terms of the physicochemical and antibacterial properties, but further studies are required to identify the optimal therapeutic range and evaluate long-term biological responses.

## Ethical approval

This study received an ethical exemption from the Health Research Ethics Committee of the Faculty of Dentistry, Universitas Trisakti (Ethical Exemption No. 791/S1/KEPK/FKG/6/2024) because it was conducted as an in vitro laboratory study and did not involve human participants or animal subjects.

## Authors contributions

NLR, FJA, and TCM were responsible for conceptualization, methodology, investigation, and drafting the manuscript. SW, and APD were responsible for conceptualization, supervision, and project administration. Moehamad Orliando Roeslan contributed to manuscript review, editing, supervision, and formal analysis. All authors have critically reviewed and approved the final draft and are responsible for the content and similarity index of the manuscript.

## Generative AI statement

The authors declare that generative AI tools were used during the preparation of this manuscript in a limited manner for language editing and for grammar correction of the manuscript. The authors take full responsibility for the content and accuracy of the manuscript.

## Source of funding

This research did not receive any specific grant from funding agencies in the public, commercial, or not for-profit sectors.

## Conflict of interest

The authors declare that they have no known competing financial interests or personal relationships that could have appeared to influence the work reported in this paper.
